# Effect of Cotton Activation and Solvent Evaporation on the Production of Regenerated Cellulose Membranes Using Lithium Chloride/*N*,*N*‑Dimethylacetamide

**DOI:** 10.1021/acsomega.6c01585

**Published:** 2026-05-29

**Authors:** Aline F. Knihs, Rita de Cassia S. C. Valle, Andrea C. K. Bierhalz, Cintia Marangoni

**Affiliations:** † Department of Chemical Engineering and Food Engineering, 28117 Federal University of Santa Catarina, Campus Universitário Reitor João David Ferreira Lima, Zip Code, 88040-900 Florianópolis, SC, Brazil; ‡ Department of Textile Engineering, Federal University of Santa Catarina, Rua Marechal Rondon 880, Zip Code, 89065-200 Blumenau, SC, Brazil

## Abstract

Cellulose is a renewable biopolymer with great potential for sustainable applications, but its high crystallinity hinders dissolution in conventional solvents. Regenerated cellulose membranes were developed from commercial cotton using the lithium chloride/N,N-dimethylacetamide (LiCl/DMAc) system. Different activation strategies (DMAc and DMAc/KMnO_4_) and DMAc evaporation times (48 and 72 h) before regeneration were evaluated. The activations did not alter the chemical structure of the cotton fibers but promoted greater dissolution efficiency, especially with KMnO_4_, without significantly affecting crystallinity. However, the degree of polymerization decreased from 2475 DP in the raw material to approximately 880 DP in the membranes obtained from both activated samples. Extending the DMAc evaporation time from 48 to 72 h improved microstructural homogeneity and increased the maximum degradation temperature by up to 44 °C. Structural analyses confirmed the transformation of native cellulose I into a predominantly amorphous regenerated structure with minor contributions of cellulose II. The results demonstrate that the combined control of cellulose activation and solvent evaporation is an effective strategy to tailor the structural and physicochemical properties of regenerated cellulose membranes, enabling their potential use in polymer-based filtration and separation applications.

## Graphical abstract

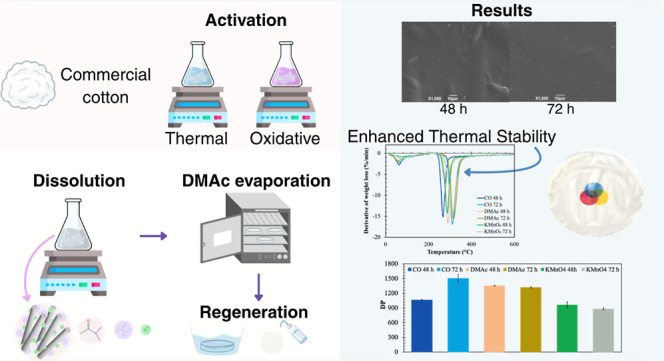

## Introduction

In the context of the growing global demand for sustainable and renewable materials, cellulose emerges as a highly promising resource due to its abundance, biodegradability, and broad availability.^1^ Cellulose can be obtained from several sources, including wood, cotton, algae, agricultural residues, and bacterial synthesis.^2^ Despite its significant advantages over fossil raw materials, the highly crystalline structure of cellulose, maintained by strong inter- and intramolecular hydrogen bonds, makes it difficult to dissolve in conventional organic solvents.^3^ Thus, cellulose undergoes thermal degradation before reaching its melting temperature, rendering traditional melt-processing routes unsuitable.^1^ Faced with these challenges, there is increasing interest in developing solvent systems that efficiently dissolve cellulose with precise control.

To overcome these limitations, several solvent systems have been explored for the dissolution of cellulose, with varying outcomes regarding the formation of cellulosic derivatives. Among the direct solvents capable of dissolving high-purity cellulose, the lithium chloride/*N*,*N*-dimethylacetamide (LiCl/DMAc) system stands out, allowing the solubilization of high molecular weight cellulose without causing chemical degradation and maintaining thermal stability.^1,2,4^ The LiCl/DMAc system is one of the most effective solvents in breaking the intermolecular interactions present in the crystalline regions of cellulose. The proposed mechanism for dissolution involves the formation of a cationic complex [Li­(DMAc)_*x*_]^+^ and the replacement of the inter- and intramolecular hydrogen bonds of cellulose by Cl^–^ ions, promoting the separation of the polymer chains of cellulose.^4–8^

However, the efficiency of cellulose dissolution in this system is strongly dependent on the intrinsic properties of the raw material, such as degree of polymerization (DP) and crystallinity, as well as on processing conditions. Cellulose sources that have high molecular weight and crystallinity, such as cotton, can also exhibit significant resistance to dissolution.^9^ In this context, activation pretreatments have been proposed as effective strategies to improve solvent accessibility by relaxing the cellulose structure, removing residual moisture, and facilitating the penetration of solvent molecules into crystalline domains.^7^ Thermal activation in DMAc has been reported to promote swelling and physical rearrangement of cellulose chains without altering their chemical structure, while oxidative treatments can induce partial depolymerization and structural opening.^9–11^ In this study, KMnO_4_ was selected as an oxidative agent due to its simplicity, low cost, and ability to promote heterogeneous oxidation without requiring catalysts or strict pH control, unlike TEMPO-mediated or periodate oxidation.^12–15^ This makes it a practical and scalable alternative for enhancing cellulose accessibility in LiCl/DMAc systems.^10,14,16–19^

In addition to exploring different activation strategies, this study also assessed DMAc removal by varying the evaporation time before regeneration. Residual DMAc must be effectively eliminated, as it can adversely affect the regenerated membranes’ mechanical, thermal, and functional properties, restricting their suitability in applications such as effluent filtration.^20^ Controlled solvent evaporation before coagulation has been shown to influence polymer chain rearrangement, porosity development, and interfacial characteristics of regenerated cellulose films.^20,21^ However, systematic investigations addressing the interplay between activation strategy and solvent evaporation time are still scarce.

Although cellulose activation has already been studied,^16–18,22^ the use of cotton combined with KMnO_4_ activation for dissolution in LiCl/DMAc remains insufficiently explored, particularly regarding the combined and systematic evaluation of oxidative activation using KMnO_4_ and controlled solvent evaporation on membrane formation and resulting structure–property relationships.^10,19^ Therefore, this study aimed to investigate how KMnO_4_ activation and processing conditions, particularly solvent evaporation, affect the properties of regenerated cellulose membranes obtained from highly purified cotton. From an application perspective, and with an emphasis on sustainability in the textile sector, such membranes could be produced from cotton textile waste and explored for wastewater treatment, especially for the removal of dyes and heavy metals. The use of purified cotton enables the assessment of the effect on the cellulose matrix without interference from dyes, finishes, or other contaminants typically present in textile residues.

## Materials and Methods

### Materials

The regenerated cellulose membranes were obtained from 100% absorbent commercial cotton wool (Cremer, bleached with hydrogen peroxide (H_2_O_2_) and free of any optical brightener, suitable for cosmetic and medical purposes). For the cellulose activation and dissolution step, *N*,*N*-dimethylacetamide (DMAc, P.A., CAS n° 127-19-5, Neon), lithium chloride (LiCl, P.A., CAS n° 7447-41-8, Dinâmica), and potassium permanganate (KMnO_4_, P.A., Proc9 Industria Química) were used. The regeneration process was performed with acetone (P.A., CAS n° 67-64-1, Êxodo científica) and deionized water. The solvents used in the characterization analyses included copper­(II) ethylenediamine solution (CUEN, Sigma-Aldrich, USA) and ethanol (P.A., CAS n° 64-17-5, Dinâmica).

### Activation of Cellulose

The thermal activation was performed by adding 0.2 g of dry commercial cotton to 20 mL of DMAc, maintained under stirring in an oil bath at 120 °C for 1 h.^11^ For the oxidative process, 1 mg of KMnO_4_ was mixed with 20 mL of DMAc, maintaining the same amount of cotton, time, and temperature conditions. After activation, the cotton was washed with distilled water, followed by washing with 4% hydrogen peroxide to remove residual manganese species, and then washed again with distilled water until neutral pH was reached.^10^

### Dissolution and Regeneration of Cellulose

For cellulose dissolution, a solution was prepared by dissolving 10% LiCl (w/w) in DMAc under stirring at 30 °C for 24 h. The cotton sample (0.2 g) without pretreatments and the two types of activated commercial cotton were dried at 60 °C for 12 h and then added to 15 mL of 10% LiCl/DMAc solution. The mixture was magnetically stirred for 3 h at 120 °C, followed by 44 h at room temperature.^11^ The progress of cellulose dissolution was monitored by optical microscopy using a Zeiss AxioLab.A1 microscope (Germany). Images were captured at a magnification of 5× to ensure a representative assessment of the fibers’ disintegration.

After dissolution, 15 mL of the cellulose solution was poured into each 8 cm diameter Petri dish and subjected to DMAc evaporation at 60 °C. Subsequently, the cellulose was regenerated in a coagulation bath with 100 mL of acetone, where it remained for 3 h. After regeneration, the membranes were washed with distilled water and dried at room temperature.

### Degree of Polymerization

The DP of the cotton fibers and regenerated films was determined in triplicate using independent membranes.^23^ The membrane samples to be tested were added to 17.5 mL of deionized water and 17.5 mL of 1 M copper­(II) ethylene-diamine solution (CUEN) in a flask containing sphere glasses. The system, under a nitrogen atmosphere, was stirred in an orbital shaker (NL-343-01, New Lab) for 3 h at 150 rpm and a temperature of 25 °C. After this period, 7 mL of the mixture was transferred to the viscometer Cannon–Fenske n° 75, which was kept in a water bath at 25 °C, to measure the flow time. The DP of the fibers and films was determined by eq 1.

1 DP=(η)×190

In eq 1, [η] is the intrinsic viscosity obtained by plotting log [(ηrel-1)/c] against c and extrapolating the straight line through the points to c = 0. Relative viscosity [ηrel] by the ratio of outflow times of the CUEN-cellulose solution (t) and the outflow time of the CUEN solution (t_0_).

### Thickness

The thickness of the membranes was determined using a digital micrometer with 0.001 mm resolution (Digimess, Brazil). At least three different membranes were evaluated, with 15 random readings performed for each membrane sample.

### Surface Hydrophobicity

The surface contact angle of the membrane was measured using the sessile drop method and a goniometer (Ramé-Hart model 250-F1) equipped with image processing software (DROPimage Advanced). The size of each distilled water drop was approximately 10 μL, and three measurements for each sample were performed.

### Scanning Electron Microscopy

The surface and cross-sectional morphologies of the membranes were examined using a JEOL JSM-6390LV scanning electron microscope (Japan) operated at an accelerating voltage of 10 kV. Before imaging, the samples were mounted on supports and coated with a thin layer of gold (Leica EM SCD 500, Germany) to increase the electrical conductivity.

### X-ray Diffraction Analysis

The crystalline structure of the cotton and regenerated membranes was analyzed using a Rigaku Miniflex600 diffractometer operated at 40 kV and 30 mA. Diffraction patterns were collected within a 2θ range of 10–30°. The crystallinity index (CrI) was calculated according to the method proposed by Segal, as described in eq 2.

2 $$CrI=\frac{I_{200}-I_{am}}{I_{200}}\times 100$$

where I_200_ is the maximum peak intensity of the crystalline plane (22.6° for cellulose I and 20.2° for cellulose II) and I_am_ corresponds to the minimum diffraction intensity associated with the amorphous fraction, located between the reflections (110)/(200) and (1–10)/(110) for cellulose I and II, respectively.^24^

### Fourier Transform Infrared Spectroscopy

FTIR spectra of the cottons and membranes were collected in the ATR mode using a Cary 660 spectrometer (Agilent Technologies, USA). The spectra were recorded in the range of 4000–800 cm^–1^ with a spectral resolution of 4 cm^–1^, averaging 32 scans per sample, and acquired in the reflection mode.

### Thermogravimetric Analysis

Thermal stability of the membranes was investigated using a STA 449-F3 Jupiter analyzer (Netzsch, Germany). Approximately 10 mg of each sample was heated up to 600 °C at a constant rate of 10 °C min^–1^ under a nitrogen atmosphere, maintained with a continuous flow of 20 mL min^–1^.

### Statistical Analysis

The results generated in this work were submitted to statistical analysis, performed in the Statistica 12.0 software. Analysis of variance was performed at a 5% significance level (α = 0.05), and mean comparisons were conducted using the Tukey test with a 95% confidence interval.

## Results and Discussion

### Effect of Activation on Cellulose Dissolution

After the pretreatments with DMAc and DMAc/KMnO_4_ under heating, the dissolution of cellulose from cotton fibers in LiCl/DMAc was monitored by optical microscopy. Figure 1 presents representative optical micrographs of the cellulose suspensions after 3 h of dissolution (Figure 1a,d,g) and after 44 h of the dissolution process (Figure 1b,e,h), as well as the macroscopic appearance of the corresponding solutions after dissolution (Figure 1c,f,i).

**Figure 1:**
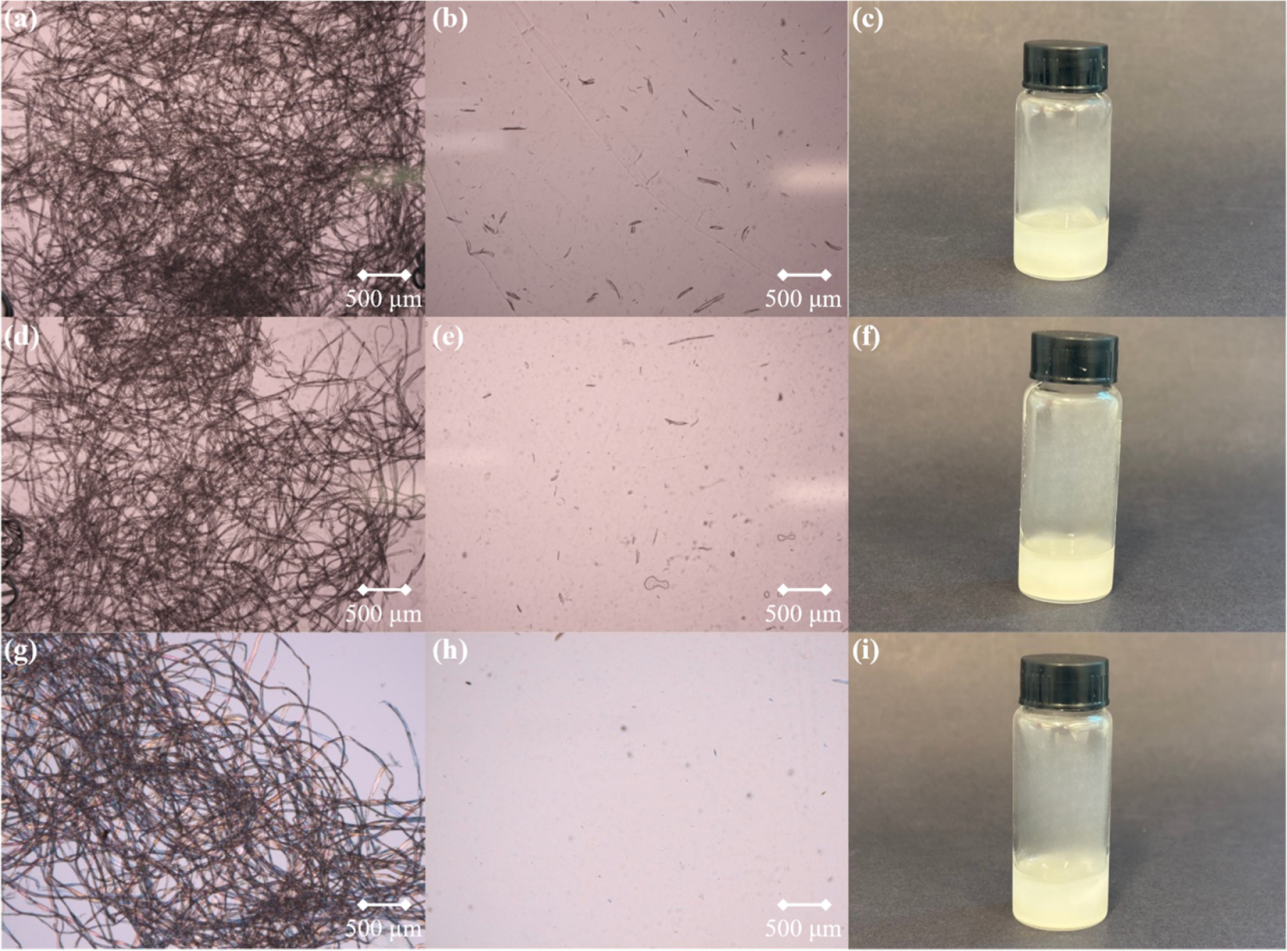
Optical microscopy images of the cotton samples at a dissolution time of 3 h: (a) without activation, (d) activation with DMAc, and (g) activation with KMnO_4_; at 44 h: (b) without activation, (e) activation with DMAc, and (h) activation with KMnO_4_; and the appearance of the solution after dissolution (c) without activation, (f) activation with DMAc, and (i) activation with KMnO_4_.

In the sample without any prior activation (Figure 1a,b), even after the total expected dissolution time, the presence of visible fibrous fragments was observed, indicating incomplete dissolution. This behavior is consistent with the high crystallinity and DP of cotton fibers, which hinder solvent penetration and chain disentanglement in the LiCl/DMAc system.^9^ In contrast, the samples subjected to thermal activation with DMAc (Figure 1d,e) showed a reduction in the amount of solid residue, indicating greater efficiency in breaking down the compact cellulose structure.

To verify whether complete dissolution could be achieved after a prolonged period, solutions without activation and with thermal activation in DMAc were agitated at room temperature for 72 h. At the end of this time, solid fragments were still identified in both samples, indicating that an extended dissolution time alone was insufficient to achieve complete solubilization. The optical microscopy images of this test are available in the Supporting Information. In the case of the sample subjected to oxidative activation with KMnO_4_ (Figure 1g,h), the resulting solution was virtually free of visible fibrous residues, suggesting complete dissolution of the cellulose. Although the optical microscopy analysis is qualitative, it provides a consistent comparative assessment of dissolution efficiency across samples, which is sufficient to support the observed trends.

Considering that the fragments observed in the dissolutions without activation and with activation in DMAc did not compromise membrane formation and that extending the dissolution time did not bring significant benefits in terms of solubilization, 44 h was established as the standard dissolution parameter, as it proved to be more efficient from a technical and operational point of view. After 44 h of dissolution, all solutions presented an opaque, viscous, and white color.

Thermal activation with DMAc promotes the opening and relaxation of cellulose polymer chains, enhancing intra- and intermolecular swelling and increasing the accessibility of hydrogen bonds, which facilitate solvent diffusion into the compact crystalline regions that are otherwise difficult to access.^25,26^ This activation aims to eliminate water adsorbed in the internal regions of the polymer matrix, favoring the efficiency of the subsequent dissolution process.^27^ It is important to highlight that this type of treatment does not promote chemical modifications in the functional groups of cellulose, which is evidenced by the absence of new bands in the FTIR spectra, as presented in Figure 2. Thus, its action is strictly physical, promoting structural and morphological changes without compromising the chemical integrity of the polymer chain.^28^

**Figure 2:**
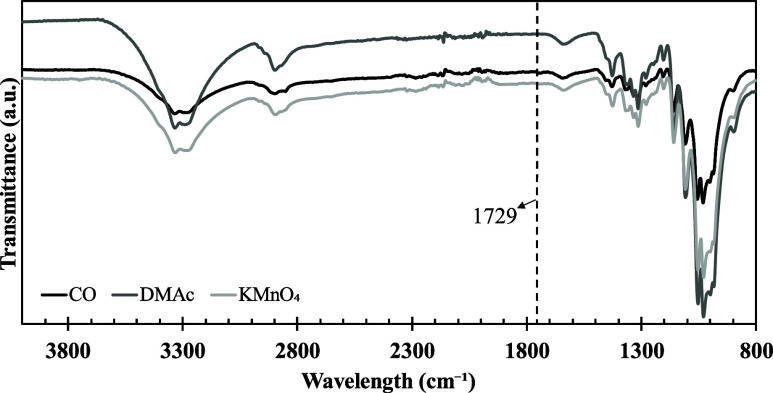
FTIR spectra of commercial cotton without activation (CO), cotton activated with DMAc, and cotton activated with KMnO_4_.

Activation with KMnO_4_ occurs through an oxidation reaction that alters the molecular structure of cellulose. This reaction is considered heterogeneous due to the structural nature of cellulose and occurs preferentially in the reducing terminal groups, contributing to the opening and modification of the polymer chains.^17^ The chemical reaction that describes the oxidation process of cellulose by the action of KMnO_4_ is represented by eq 3.^16,29,30^

3 $$cell-OH+KMnO_{4}\to cell\cdot +MnO_{2}^{-}+{OK}^{+}$$

In this redox system, permanganate acts as a strong oxidizing agent where Mn­(VII) is reduced to lower oxidation states, such as Mn­(IV) in the form of MnO_2_. This process generates free radicals on the cellulose chain (cell^•^), initiating the oxidative opening of the rings. Cellulose oxidation can introduce additional functional groups, such as carboxylic acid, increasing the density of negative charges on the macromolecule’s surface and, consequently, favoring its interaction with polar solvents and improving solubility in systems such as LiCl/DMAc.^18,31^ The oxidation preferentially occurs at the hydroxyl groups of the cellulose backbone, particularly at the secondary hydroxyls located at C2 and C3, while the primary hydroxyl group at C6 is also susceptible to oxidation, typically leading to the formation of carboxyl groups under stronger oxidative conditions. It is important to note that the – CH_2_OH group at the C6 position is inherently present in native cellulose and is not formed during oxidation but rather can be transformed into other oxidized functionalities.^18^

The formation of these new groups could be present in FTIR spectra, specifically in the absorption at 1729 cm^–1^, which is associated with the stretching vibration of the carbonyl group, which may belong to aldehydes, ketones, or carboxylic acids.^32^ However, the FTIR spectra of untreated cotton (CO), DMAc-activated cotton, and KMnO_4_-activated cotton (Figure 2) showed no significant differences, indicating that the treatments did not introduce detectable changes in the main functional groups of the cellulose. According to Zhang et al.,^17^ this behavior may be related to the low absorption intensity in the 1729 cm^–1^ region. Furthermore, the oxidation of cellulose by KMnO_4_ leads to the formation of hydrated hemialdehyde groups, which do not present a characteristic stretching band of the aldehyde group.^33^ Therefore, the absence of clear FTIR evidence does not exclude the occurrence of oxidation but rather suggests that the degree of modification is low or involves functional groups with limited infrared activity, highlighting the intrinsic limitations of the technique.

XRD analysis (Figure 3) revealed that the native crystalline form of cotton cellulose I was preserved after activation treatments with DMAc and KMnO_4_. The diffraction patterns of the samples exhibited the typical reflections of cellulose I, with a sharp crystalline peak at 2θ = 23.2° attributed to the (200) plane, accompanied by two broader peaks at 14.8° (−110) and 16.5° (110).^34,35^ The calculated crystallinity index for the cotton samples was 70.6% for the untreated cotton, while thermal activation with DMAc slightly reduced this value to 66.9%, and activation with KMnO_4_ resulted in 69.2%. These results demonstrate that both treatments caused only minor variations in crystallinity, indicating that the activation processes did not compromise the native crystalline arrangement of cellulose, corroborating the FTIR results.

**Figure 3:**
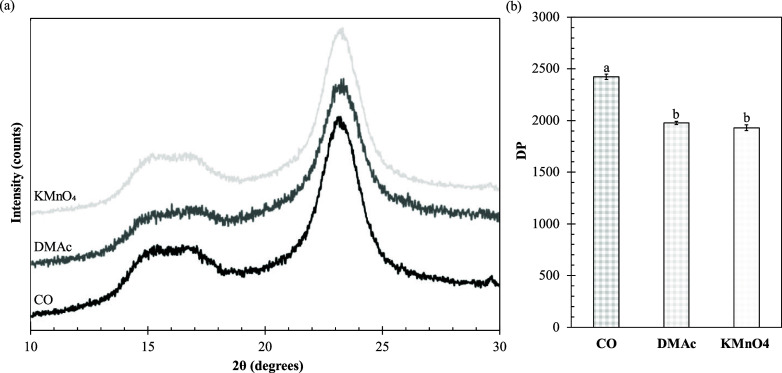
XRD (a) and DP (b) analysis for cotton without activation, cotton activated with DMAc, and cotton activated with KMnO_4_. In (b), columns with the same letter indicate no significant differences (*p* < 0.05) by the Tukey test.

Furthermore, the DP values presented in Figure 3b elucidate the effects of activation. The nonactivated cotton exhibited a DP of 2475 ± 88, while DMAc-activated cotton showed a reduced DP of 1977 ± 15, and KMnO_4_-activated cotton exhibited a DP of 1930 ± 28. Both activation methods promoted a significant reduction in the DP in relation to the original cotton due to the subjection to high temperature, the time of occurrence, and the presence of solvents.^36^ This reduction indicates that the applied activations resulted in the partial depolymerization of the cellulose chains. However, the values obtained remain higher than the DP of microcrystalline cellulose (DP≅250)^37,38^ and compatible with the DP of cotton fabrics (DP = 2200)^39^ and with bleached Kraft wood pulp (DP = 1900).^40^

### Influence of DMAc Evaporation

After dissolution of the cotton cellulose, the influence of DMAc evaporation time before regeneration on membrane formation was evaluated by drying the cast solutions at 60 °C for 48 and 72 h. This step plays a critical role in controlling polymer chain mobility, solvent retention, and the subsequent phase inversion process during regeneration, directly affecting membrane morphology and properties. The macroscopic appearance of the cellulose membranes regenerated after DMAc removal for 48 or 72 h is demonstrated in Figure 4.

**Figure 4:**
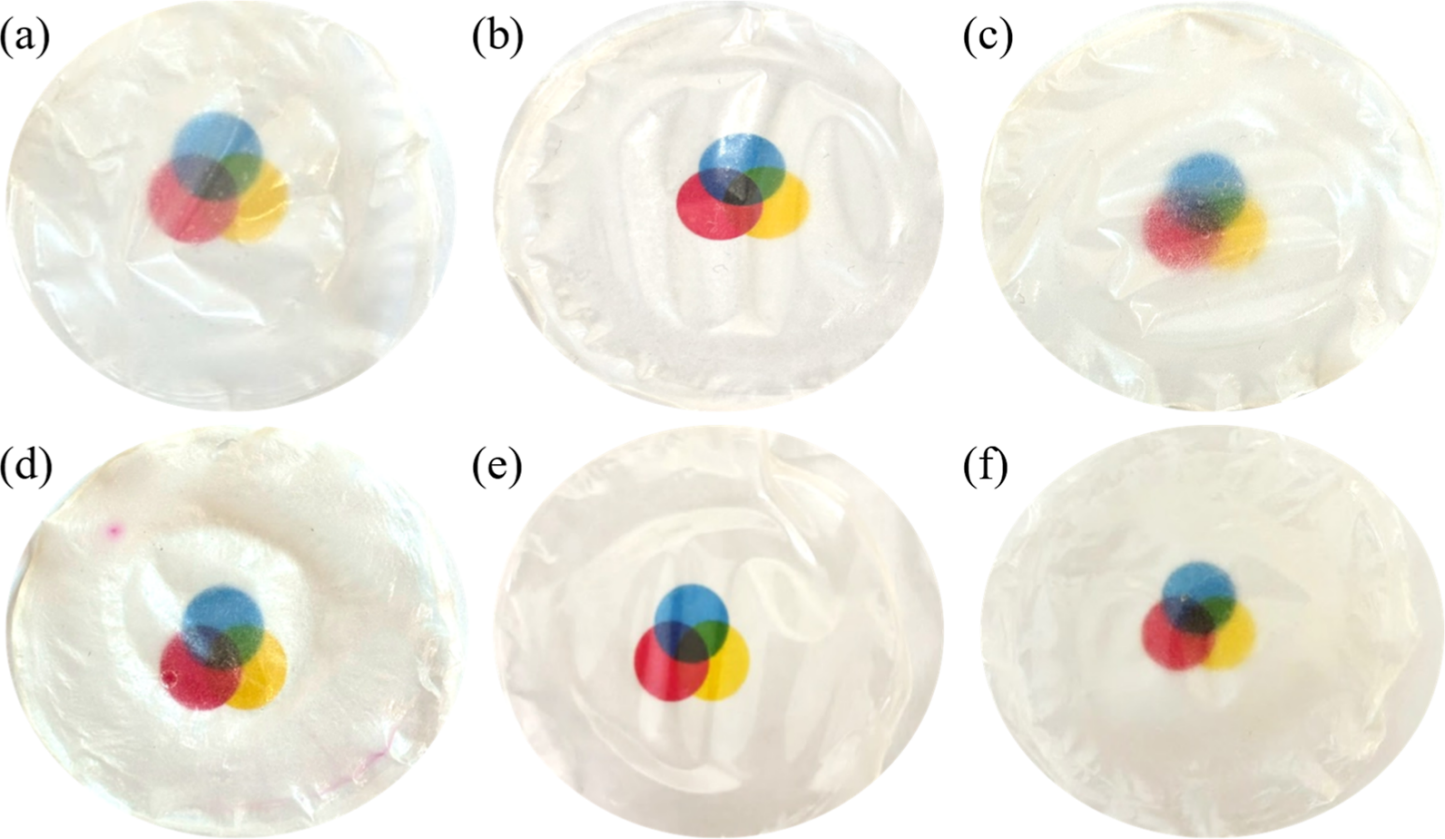
Visual appearance of membranes obtained from cellulose without activation (a,b), activated with DMAc (c,d), and activated with KMnO_4_ (e,f), after evaporation of DMAc at 60 °C for 48 h (a, c, e) and for 72 h (b, d, f).

In general, the membranes presented a translucent appearance and uniform surface structure regardless of the evaporation time and the applied pretreatment. It is noteworthy that the characteristic KMnO_4_ coloration was eliminated after washing with H_2_O_2_, without interfering with the translucent appearance of the membrane obtained from the KMnO_4_-activated cotton. The DMAc removal was apparently effective at both times evaluated, 48 and 72 h, demonstrating no significant visual impact on the homogeneity or transparency of the membranes. It is important to note that visual assessment of transparency may be influenced by factors such as thickness and light scattering and therefore should be interpreted qualitatively.

Evaporation for 24 h had been previously tested, but it was insufficient for the complete removal of DMAc from the membrane. This result suggests that under the conditions evaluated, the type of activation and evaporation time of DMAc exerted a limited influence on the final appearance of the membranes, with a more pronounced effect only in one sample.

### Physicochemical Properties of Membranes

The DP of the regenerated samples was evaluated by viscometry, and the results are presented in Table 1. The results indicate that the type of activation had a statistically significant effect on the DP (*p* < 0.05), leading to distinct extents of polymer chain depolymerization. The highest DP values were observed in the unactivated samples (CO 48 and 72h), followed by those activated with DMAc. Although KMnO_4_ activation was effective in promoting solubilization, it resulted in the most pronounced depolymerization of the polymer chains. In contrast, variations in the evaporation time did not significantly influence the DP for any of the samples under the conditions evaluated.

**Table 1:** Degree of Polymerization, Thickness, and Contact Angle of Membranes Under Different DMAc Evaporation Conditions^a^

sample	DP	thickness (μm)	contact angle (deg)
CO 48 h	1458 ± 37a	65.15 ± 8.68a,b	38.86 ± 2.92b
CO 72 h	1505 ± 78a	54.36 ± 5.40b	63.80 ± 8.74a
DMAc 48 h	1355 ± 06b	62.27 ± 2.34a,b	32.49 ± 3.41c
DMAc 72 h	1321 ± 15b	66.08 ± 6.22a	41.28 ± 6.88b
KMnO_4_ 48h	965 ± 61c	58.85 ± 5.03a,b	57.43 ± 5.62a
KMnO_4_ 72 h	883 ± 23c	66.86 ± 4.82a	56.83 ± 3.76a

a Averages with the same letter in the same group column indicate no significant differences (*p* < 0.05) by the Tukey test.

The reduction in DP from approximately 2400 (cotton) to approximately 1300 (regenerated membrane) cannot be attributed solely to the activation step at 120 °C since Figure 3b shows minimal changes after this treatment. Instead, the decrease can be attributed to the combined effects of dissolution and regeneration in the LiCl/DMAc system. Prolonged exposure to elevated temperatures during dissolution, along with solvent–polymer interactions, may promote chain scission and contribute to depolymerization. This interpretation is consistent with the findings of Ono et al. (2018),^41^ who reported that significant depolymerization can be minimized only when the entire process is conducted at room temperature. Therefore, the observed decrease in DP, including that of the untreated sample, is attributed to the combined effects of thermal exposure and processing conditions and not just to the activation step.

The DMAc evaporation time and the interaction between the activation type and the evaporation time did not show a statistically significant effect (*p* > 0.05), suggesting that the variation in the evaporation time does not exert a relevant influence on the DP under the conditions evaluated.

The membrane thickness varied among the samples, although the same nominal volume of solution was cast into Petri dishes. This variation can be attributed to differences in the solvent evaporation rate, polymer chain organization, and possible shrinkage during drying. Longer evaporation times may promote increased structural rearrangement and densification, leading to thickness variations even under nominally identical casting conditions.^42^ Therefore, the thickness was not directly controlled as an independent parameter but is a result of the processing conditions.

Hydrophilicity plays an important role in membrane applications, especially in wastewater treatment. This property can be influenced by several factors, including surface roughness, porosity, surface heterogeneity, or the liquid sorption capacity of the polymer matrix.^43^ In addition, the orientation of the cellulose molecular chains on the film surface can also exert a significant influence on the membrane hydrophobicity.^44^ In the present study, all membranes exhibited contact angles below 90°, confirming their inherently hydrophilic nature, despite variations in wettability among the samples. The measured water contact angles showed statistically significant differences, as summarized in Table 1.

According to the Tukey test, the activation with DMAc with 48 h of evaporation resulted in a significantly lower contact angle value than the other samples. On the other hand, the highest contact angles were observed in the membranes without activation (72 h) and in those activated with KMnO_4_ (48 and 72 h). These differences can be attributed to variations in surface structure, chain orientation, and cellulose rearrangement during solvent evaporation and regeneration. Such structural changes may lead to slight increases in contact angle due to denser packing or reduced availability of polar groups at the surface; however, they do not result in a transition to hydrophobic behavior.^45–47^ This behavior is consistent with that of commercial regenerated cellulose membranes, which are typically hydrophilic and exhibit water contact angles generally ranging from approximately 20° to 70°, depending on processing conditions and surface characteristics.^43,44,48–50^

### Morphology Analysis by SEM

The effect of activation pretreatments and the DMAc evaporation conditions on the surface morphologies of the regenerated membranes was analyzed by scanning electron microscopy (SEM), and the micrographs are presented in Figure 5.

**Figure 5:**
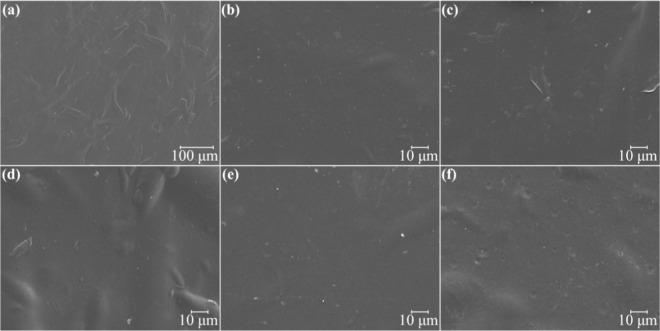
SEM images of the membrane surfaces obtained: (a) without activation, 48 h of evaporation; (b) without activation, 72 h of evaporation; (c) activation with DMAc, 48 h of evaporation; (d) activation with DMAc, 72 h of evaporation; (e) activation with KMnO_4_, 48 h of evaporation; and (f) activation with KMnO_4_, 72 h of evaporation.

The membrane without activation and subjected to evaporation for 48 h (Figure 5a) presented a rough, heterogeneous, and wrinkled surface, which agrees with its opaque visual appearance. These features suggest nonuniform solvent removal and heterogeneous polymer chain packing, resulting in surface irregularities. The membranes activated with DMAc and KMnO_4_ evaporated for 72 h (Figures 5d,f, respectively) also presented slightly wrinkled surfaces. This morphology is attributed to partial entrapment of DMAc within the polymer matrix, caused by incomplete solvent evaporation, followed by rapid coagulation in acetone, which induces surface shrinkage and wrinkling during regeneration.^21^

On the other hand, membrane samples without activation and 72 h evaporation (Figure 5b), activated with DMAc (Figure 5c), and with KMnO_4_ (Figure 5e), both with 48 h of evaporation, exhibited more homogeneous surfaces. The membrane samples without activation and 72 h evaporation and activated with KMnO_4_ with 48 h of evaporation also presented better transparency, as demonstrated in Figures 1b,e, respectively. These results demonstrated that the efficiency of DMAc removal depends on both the evaporation time and the membrane structure. Although longer evaporation times generally favor solvent removal, the formation of denser or less uniform structures can hinder the complete solvent diffusion. This explains why the relationship is not strictly linear and why some samples do not show a direct improvement in solvent removal at longer evaporation times.

These findings are in agreement with those of Yun et al. (2008) and Nayak et al. (2008),^20,21^ who observed similar effects of controlled evaporation on the morphology and surface properties of the membranes. Overall, the results suggest that the combination of activation type and DMAc evaporation time influences not only the physical aspect but also the morphology and, consequently, the final properties of the membranes.

### Structural Characterization by X-ray Diffraction

XRD analysis was performed to investigate the crystalline structure of the membrane after the dissolution and regeneration processes. Figure 6 shows the patterns of the membranes obtained under different activation and evaporation conditions of DMAc.

**Figure 6:**
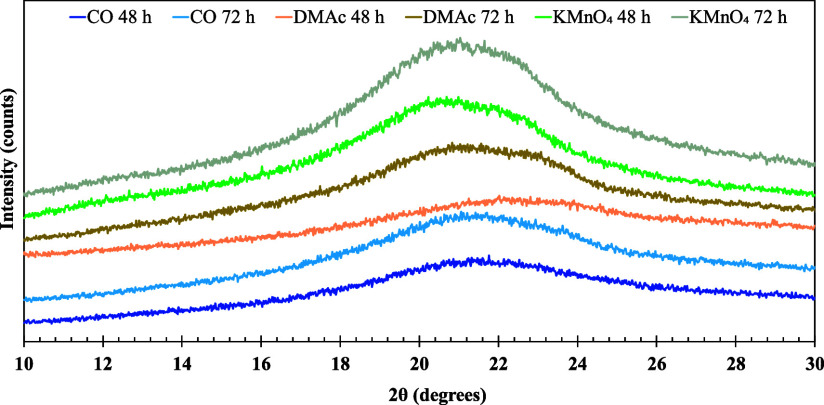
XRD analysis for activated cotton and regenerated cellulose membranes prepared under different activation and DMAc evaporation conditions.

After the dissolution and regeneration process, a shift in the diffraction peaks of the membranes (Figure 6) was observed, and a broad peak at 20.9° (101) and 21.6° (020) was observed, indicating the conversion of the crystalline form of cellulose I to an amorphous structure, with the presence of a small fraction of cellulose II.^44,51^ This transformation occurs because, during the dissolution process with LiCl/DMAc, the inter- and intramolecular hydrogen bonds are broken, disrupting its original crystalline organization. Subsequently, in the regeneration process, the cellulose is rapidly precipitated. This process is so fast that the hydrogen bonds do not have enough time to reorganize into an ordered crystalline structure, thus resulting in the formation of a predominantly amorphous structure.^51^

The diffractograms obtained revealed variations in peak intensity and width among the samples, reflecting structural differences induced by the preparation conditions. The membranes activated with KMnO_4_, especially the one subjected to 72 h of evaporation, exhibited more intense and defined peaks, suggesting a greater degree of structural order. Similarly, the membranes without activation (48 and 72 h) as well as the sample activated with DMAc and evaporated for 72 h also presented sharper reflections, consistent with a partial reorganization of the regenerated cellulose structure.

In contrast, the sample activated with DMAc and evaporated for only 48 h presented broader and lower-intensity peaks, indicative of a strongly amorphous morphology.^52^ These results reinforce that both the evaporation time and activation type significantly influence the molecular reorganization of cellulose during the regeneration process. Longer evaporation times appear to favor the formation of more organized regions, possibly because of the gradual elimination of the solvent and the rearrangement of the polymer chains in a less dynamic environment.

### Identification of Functional Groups by Fourier Transform Infrared Spectroscopy

FTIR spectroscopy was used to analyze the changes in the chemical structures of the membranes obtained under different evaporation conditions of DMAc, as shown in Figure 7. The broad region between 3700 and 3000 cm^–1^ was attributed to the stretching vibrations of the O–H bonds, resulting from the inter- and intramolecular hydrogen interactions in the cellulose chains.^44^ The peak around 2900 cm^–1^ corresponds to the stretching of the C–H bonds. The vibration at 1640 cm^–1^, present in all samples, is related to the vibration of the adsorbed water molecules. The intense band at 1027 cm^–1^ is attributed to the characteristic stretching of the C–O–O bonds.^35^

**Figure 7:**
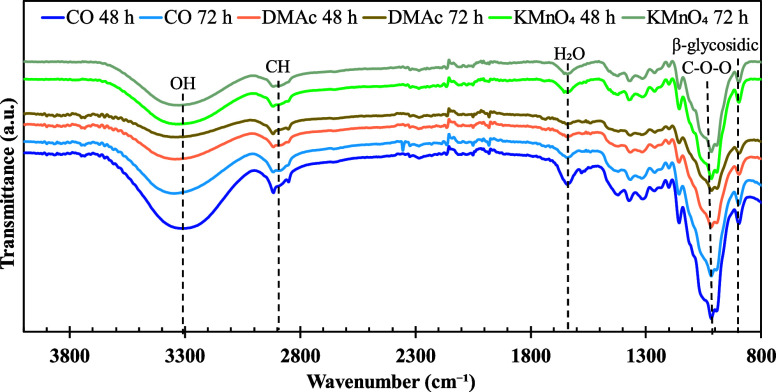
FTIR spectra of membrane samples under different DMAc evaporation conditions.

Compared with activated cotton (Figure 2), the peak around 1430 cm^–1^, attributed to CH_2_ symmetric bending, showed reduced intensity and shift in the membranes. The small band in the membrane samples at 894 cm^–1^ is associated with the β-glycosidic bond of cellulose, which represents the amorphous region of cellulose.^51,53^ These bands confirm the conversion of cellulose I to a predominantly amorphous structure and to the type II cellulose polymorph, as verified in the XRD analysis.^38,44,51^ Additionally, the elongation of the band at 3336 cm^–1^ reflects the changes in hydrogen bonds, indicating greater structural disorder. The reduction in the intensity of the peaks at 1335, 1315, 1111, 1057, and 1033 cm^–1^ reinforces the absence of cellulose I in the membranes, confirming the predominance of amorphous cellulose.^51,54^ It is worth mentioning that no peak was observed at 1620 cm^–1^ due to amide I, verifying the removal of LiCl/DMAc during the postwash treatment.

Changes in intensity and band broadening were observed between the membranes, mainly in the region of 3336 cm^–1^, associated with hydrogen bonds. However, these variations were subtle and did not indicate the presence of new functional groups or relevant changes in the chemical composition of the membranes.

### Thermal Stability of Membranes by Thermogravimetric Analysis

The TGA and DTG curves of the membranes produced with different activations and under different DMAc evaporation conditions are presented in Figure 8. The initial weight loss, observed in all samples between 50 and 150 °C, is attributed to the evaporation of water adsorbed on the membranes.^21,55^

**Figure 8:**
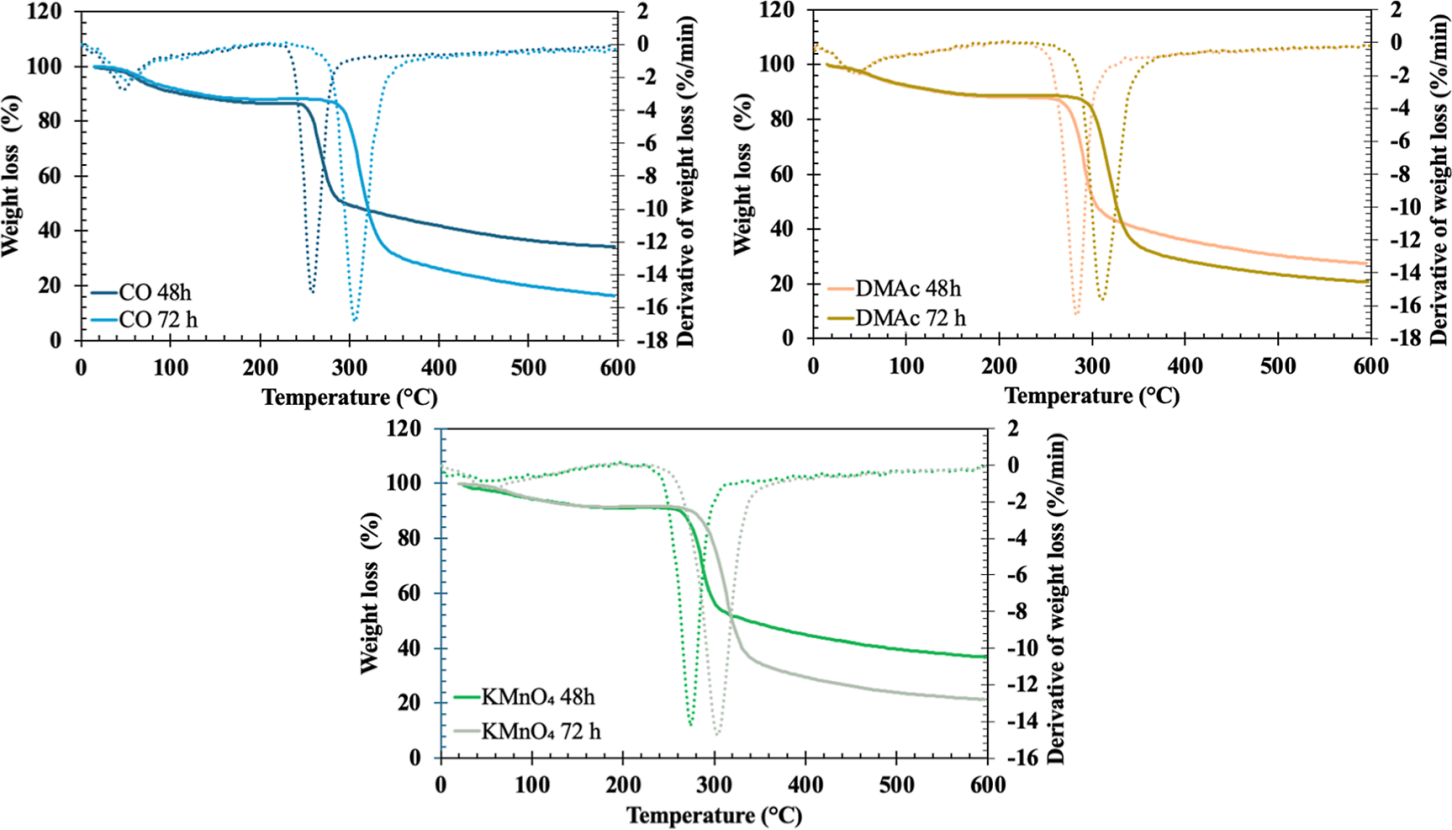
Thermogravimetric analysis (TGA) and derivative thermogravimetric (DTG) curves of regenerated cellulose membranes prepared under different activation and DMAc evaporation conditions.

The main stage of thermal decomposition of the membranes occurred in the range of 250 to 390 °C, related to the thermal degradation of cellulose, involving carbonization and volatilization processes.^44,51^ The reduction in decomposition temperatures compared to native cellulose, whose typical values of initial decomposition temperature (Tonset) and maximum decomposition temperature (Tmax) are generally between 300 and 350 °C, and with a final decomposition temperature (Tendset) close to 400 °C, is attributed to the structural transformation of cellulose I into amorphous forms and/or into cellulose II during the dissolution and regeneration process.^48,56^

The comparison between membranes with 48 and 72 h of evaporation shows that the DMAc evaporation time plays a fundamental role in the thermal stability of the regenerated membranes. The membranes subjected to 72 h of evaporation, regardless of the activation type, presented greater thermal stability, reflected in higher Tonset values (between 250 and 268 °C), with a Tendset close to 360 °C, in addition to greater mass losses and lower residue levels at 600 °C (approximately 16–21%). In contrast, samples evaporated for 48 h showed lower Tonset values (∼238–244 °C) and Tendset values around 326 °C, with considerably higher residues (27–37%). These trends, consistent across all membranes, are summarized in Table 2.

**Table 2:** Initial Degradation Temperature (Tonset), Maximum Degradation Temperature (Tmax), Final Degradation Temperature (Tend), Main Mass Loss in the Degradation Stage, and Residual at 600 °C of Membrane Samples

sample	tonset °C	Tmax °C	Tend °C	loss of mass %	residue at 600 °C %
CO 48 h	238	268	300	38	34
CO 72 h	267	312	357	56	16
DMAc 48 h	244	292	326	45	27
DMAc 72 h	268	315	360	56	21
KMnO_4_ 48 h	244	286	321	40	37
KMnO_4_ 72 h	250	313	359	57	21

These results suggest that longer evaporation times (72 h) favor more efficient solvent removal, promoting greater reorganization of the polymer chains and the formation of more ordered regions, which translates into better thermal performance. In addition to solvent removal, this behavior is also associated with structural reorganization of cellulose chains and differences in crystallinity, which contribute to an enhanced thermal resistance.

The higher residue content observed in membranes evaporated for 48 h may be associated with the presence of a residual solvent or partially degraded byproducts trapped in the polymer network, which tend to catalyze secondary degradation pathways and contribute to the formation of char. Furthermore, the DTG curves show that these samples also exhibited steeper mass loss rates, indicating that once degradation begins, it occurs more intensely and is concentrated within a narrower thermal range. This phenomenon is typical of more organized materials, whose degradation occurs abruptly after a well-defined structural threshold is breached.^44^

## Conclusions

This work demonstrates the successful production of regenerated cellulose membranes from commercial cotton using the LiCl/DMAc solvent system, highlighting the critical role of cellulose activation and solvent evaporation in controlling the membrane structure and properties. Oxidative activation with KMnO_4_ was the most effective in promoting cellulose dissolution, while thermal activation with DMAc also contributed albeit to a lesser extent. Neither activation strategy caused detectable chemical modification of the cellulose backbone, as confirmed by FTIR analysis; however, a reduction in the DP was observed after regeneration, being more pronounced for KMnO_4_-activated samples.

The DMAc evaporation time had a direct impact on the membrane properties: evaporation for 72 h resulted in greater thermal stability, less residue, and a better surface uniformity. Structural analyses confirmed the regeneration of cellulose into an amorphous structure, and wettability tests showed that the hydrophobicity can be adjusted according to the preparation conditions.

Overall, the combined control of the activation strategy and solvent evaporation enables the tailoring of physicochemical properties of regenerated cellulose membranes without compromising chemical integrity.

## Supplementary Material



## Data Availability

The data underlying this study are available in the published article and its Supporting Information.

## Notes

Optical microscopy images after 72 h of dissolution of cotton (PDF)
