# Supplementary material for: Effect of Cotton Activation and Solvent Evaporation on the Production of Regenerated Cellulose Membranes Using Lithium Chloride/N,N‑Dimethylacetamide
Source: ACS Omega. 2026 May 29;11(22):32802–12. doi: 10.1021/acsomega.6c01585 (PMC13261413; doi:10.1021/acsomega.6c01585)

Effect of cotton activation and solvent evaporation on the production of regenerated cellulose membranes using lithium chloride/N,N-dimethylacetamide

Aline F. Knihs[1], **Rita de Cassia S. C. Valle**[2], **Andrea C. K. Bierhalz**[2*], **Cintia Marangoni**[1,]

[1]Department of Chemical Engineering and Food Engineering, Federal University of Santa Catarina, Campus Universitário Reitor João David Ferreira Lima, zip code 88040-900, Florianópolis, SC, Brazil.

[2]Department of Textile Engineering, Federal University of Santa Catarina, Rua Marechal Rondon 880, zip code 89065-200, Blumenau, SC, Brazil.

*Corresponding author: andrea.krause@ufsc.br

Supporting Information – SI

Figure S1 Optical microscopy images after 72 h of dissolution of cotton without activation (a) and cotton activated with DMAc (b).

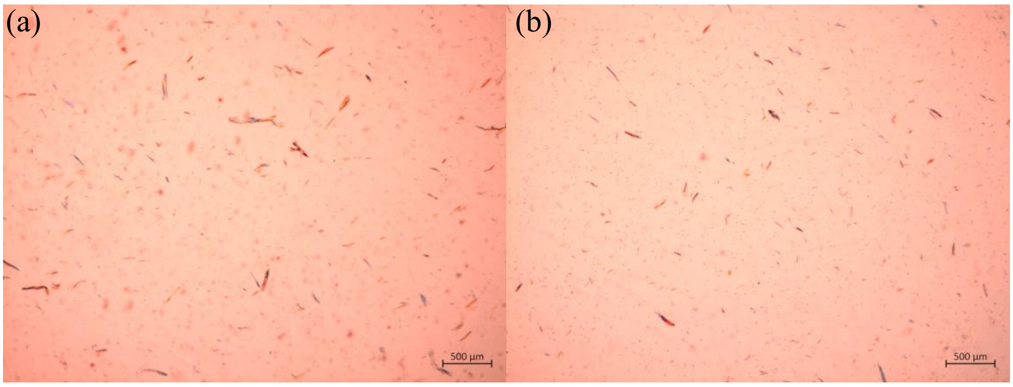

Supplement: Supplementary file 1 [file ao6c01585_si_001.pdf]
